# Disordered eating in elite youth athletes: A scoping review of studies published since 2000

**DOI:** 10.1016/j.jsampl.2023.100040

**Published:** 2023-11-01

**Authors:** Maxwell Marrows, Hilary Grover, Georgina Buckley, Nikki A. Jeacocke, Courtney C. Walton

**Affiliations:** a Eastern Health, Melbourne Australia; b Centre for Youth Mental Health, Faculty of Medicine, Dentistry and Health Sciences, University of Melbourne, Parkville, Victoria, Australia; c MP Sports Physicians, Frankston, Australia; d Melbourne, Australia; e Performance Services, Australian Institute of Sport, Canberra, Australian Capital Territory, Australia; f Elite Sports and Mental Health, Orygen, Melbourne, Australia; g Melbourne School of Psychological Sciences, Faculty of Medicine, Dentistry and Health Sciences, University of Melbourne, Parkville, Victoria, Australia

**Keywords:** Youth sports, Mental health, Feeding and eating disorders, Gender

## Abstract

**Objectives:**

The purpose of this scoping review was to explore what is known about eating disorders and disordered eating in elite youth athletes aged 12–18. This review intended to explore what is known about the prevalence, risk factors, and outcomes of these conditions.

**Design:**

Scoping Review.

**Method:**

A scoping review was conducted following the Johanna Briggs Institute (JBI) and Preferred Reporting Items for Systematic reviews and Meta-Analyses extension for Scoping Reviews (PRISMA-ScR) methodology. Six key databases were searched to identify articles for inclusion: PsycInfo, MEDLINE, CINAHL, SPORTDiscus, Scopus, and Google Scholar. Data was subsequently extracted and summarised in line with the research questions.

**Results:**

In total, 21 studies were found that met inclusion criteria. The included studies overwhelmingly focused on girls, European populations, and used a cross-sectional quantitative study design. Where clinical interview was used, there were higher rates of eating disorders among elite youth athletes compared to the general youth population. The most cited risk factors included female gender, leanness sports, and those with higher body mass index. Eating disorders and disordered eating were found to be associated with higher rates of depression and anxiety but there was limited investigation of other associated outcomes.

**Conclusions:**

Findings from this review suggest that elite youth athletes are at risk of eating disorders and disordered eating. However, significant limitations in the field exist and further research is needed using clinical interview and population specific screening tools to better understand the prevalence, risk factors and outcomes of disordered eating and eating disorders to support this population.

## 1 Introduction

Considering the pervasive pressures to conform to specific body compositions, sports environments may contribute to the development of unhealthy eating behaviours and cognitions among athletes [1,2]. Consequently, eating disorders (EDs) and disordered eating (DE) have become of significant concern within elite sport [2]. The vulnerability of younger athletes is particularly relevant, given that youth is a high risk developmental period for the emergence of mental health disorders [3], with EDs frequently first showing their onset during adolescence [4,5]. Indeed, EDs are thought to be prevalent among approximately 6–8% of young people generally [6]. Critically however, not all youth sports contexts are equal, and individuals engaged in more highly competitive sport may be at particular risk [7,8].

Elite youth athletes (EYAs) can be distinguished from youth engaged in recreational sport, as being primarily performance-focused, often at the expense of critical psychosocial and educational experiences, and with a goal of progression to elite adult sport [9]. This is because in contrast to recreational settings where sport is generally considered beneficial to mental health, EYAs face a combination of age-related developmental risks, in combination with high performance sport-specific risk factors [5,10]. As described in depth elsewhere [9], EYA's face increasing pressure to perform and intensive training regimes, potentially leading to an increased desire to manipulate body weight or shape [[10], [11], [12]]. Thus, enhancing our understanding of how EDs and DE exist within elite youth sport is critical for advancing clinical care and creating healthy sports environments.

In the current review, we consider EDs as those which can be classified by diagnostic criteria (e.g., the Diagnostic and Statistical Manual of Mental Disorders), and sit at one end of a spectrum of eating behaviour [2]. The opposite end of this spectrum has been described as optimised nutrition [2] and includes intuitive eating and body appreciation [[13], [14], [15], [16]]. DE, a more recently described concept, is thought to exist just below EDs within this spectrum. DE is subclinical, but includes symptoms such as skipping meals. Individuals, including athletes, may move along this spectrum throughout their lives or careers. As a result, the ever-increasing understanding of disordered eating may be helpful in the prevention and early identification of eating disorders [2,17].

To our knowledge, no systematic summary of EDs and DE among EYAs exists in the literature. Therefore, we conducted a scoping review to collate what is currently known about this topic. Critically, this review focused on EYAs only, given the aforementioned differences among this population to recreational youth sports [9]. Specifically, we aimed to describe and summarise: (a) prevalence, (b) risk factors and (c) outcomes pertaining to EDs and/or DE among elite youth athletes.

## 2 Methods

This review was conducted in accordance with the JBI scoping review methodology and following PRISMA-ScR reporting guidelines [18]. A preliminary search of various databases including Cochrane Database of Systematic Reviews, JBI Evidence Synthesis, Open Science Framework and Google Scholar was completed at the outset of this work and no protocols for relevant reviews were found by the first author (MM). The protocol for this scoping review was formally registered with Open Science Framework (OSF) on 28/09/2022 ​at https://osf.io/exa8k/. This review was originally undertaken by author MM in the form of a Master of Psychiatry research thesis at the University of Melbourne, supervised by the senior author (CW).

### 2.1 Eligibility criteria

This scoping review included English language, peer-reviewed studies published from the year 2000 or later. The year 2000 was chosen as an arbitrary yet pragmatic cut-off, with the goal to include only the most current and relevant literature, given the ongoing rapid professionalisation of youth sport and subsequent limitations of comparison across large time periods. This review defined youth as those aged between 12 and 18. We chose this age range for several reasons. First, highly competitive and demanding sports participation prior to the age of 12 is considered *early* specialisation and may be harmful [19]. At the older end of the spectrum, professionalism and adult competition often occurs after the age of 18 ​at which point athletes are no longer in youth contexts. Therefore, acknowledging that no universally accepted applied definition of youth athletes exist [9], this review focuses on athletes aged 12–18. Similarly, though definitions of elite in youth sport remain elusive, ‘elite’ was defined using the broadest criteria proposed by Swann, Moran [20]. Youth athletes competing in international, national, and regional competitions as well those participating in high performance talent development programs were considered elite. Studies were only included if they specifically addressed the prevalence, risk factors, or outcomes of EDs or DE. Specific inclusion and exclusion criteria are available within the supplementary materials and in our pre-registered protocol.

### 2.2 Search strategy

Articles were collected from five databases: PsycInfo, MEDLINE, CINAHL, SPORTDiscus, and Scopus. A Google Scholar keyword search was also performed as a supplemental measure with the first 200 results screened. The search strategy (see supplementary materials) was developed in consultation with a University of Melbourne librarian specialised in the conduct of systematic searches. Search terms were modified according to the specific requirements of each database by including Medical Subject Headings (MeSH) terms where available, in addition to the extensive keyword search.

### 2.3 Screening

**Fig. 1:**
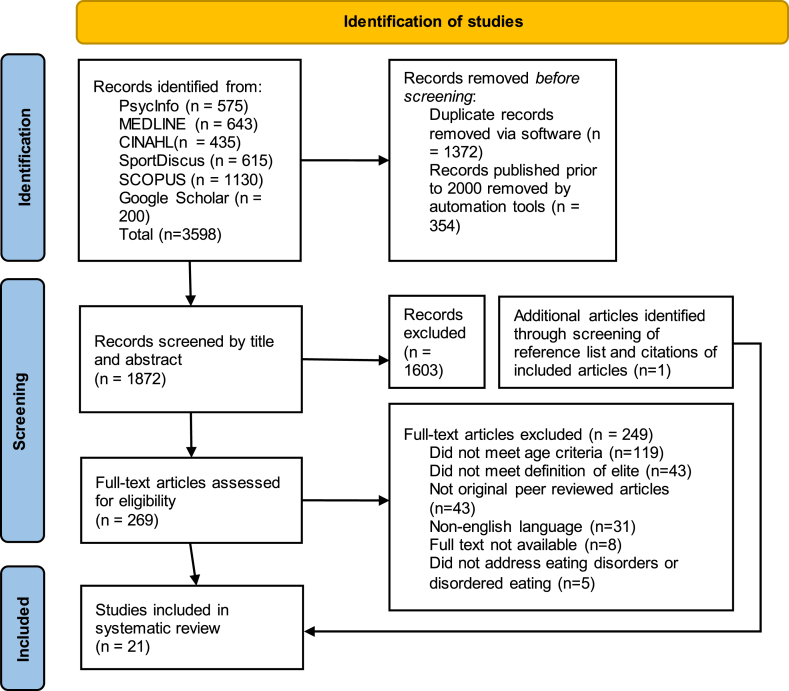
Prisma flow diagram depicting the movement of information throughout the scoping review. It plots the databases searched, number of records screened by abstract and full text and the reasons for exclusion.

The initial search was completed on 01/10/2022 and yielded 3598 citations. After duplicates and articles published prior to 2000 were removed using the software Endnote 20 and Covidence, there were 1891 articles that proceeded to screening. Titles and abstracts were initially screened in duplicate by two reviewers (MM & HG) with Covidence review software against the inclusion and exclusion criteria described in Appendix 1. There were 1621 articles which did not meet inclusion criteria and were discarded, with 270 articles proceeding to duplicate full text screening by two reviewers (MM & HG). Any disagreements at either stage were resolved by consensus. Forward and backward screening through reference lists and citations for all included articles was conducted by one reviewer (MM) between 11/03/2023 and 18/03/2023 with one additional article identified. This process is depicted in Fig. 1 in the form of a PRISMA flowchart.

### 2.4 Charting the data

Data extraction was performed by author MM using a Microsoft Excel and a data extraction tool published with the original protocol. Data extracted included information regarding author/s, year, journal, study design, research objectives, sample size, location, participants, concept, context, and key findings relevant to the review questions. The senior author (CW) reviewed 20% of the data extraction forms for quality assurance with three small discrepancies found. Only one of these resulted in a change to the data; a single study was corrected to label participants as from both team and individual sports, rather than just team sports.

## 3 Results

### 3.1 Sample and study characteristics

Table 1, Table 2 summarise all 21 papers included in this review. Table 1 includes key demographic information and Table 2 includes study measures, prevalence, risk and protective factors, and outcomes of EDs. Four of the included studies (19%) were published between 2000 and 2009, 13 studies (62%) were published between 2010 and 2019 and a further four studies (19%) had been published since 2020. Studies were primarily conducted in Europe (52%), while three (14%) were from Brazil and two (10%) were from the USA. Nine studies included both male and female-identifying genders, 11 (52%) included only girls, with just one study reporting on only boys [21]. No studies reported data on any other genders. The median samples size was 220 with the smallest sample 23 participants [22] and the largest including 1138 participants [10]. Thirteen studies (62%) included both team and individual sports, five (24%) included only team sports, and three (14%) included only individual sports. Seven studies (33%) included athletes attending elite sport schools in Europe and at least 15 (71%) included athletes competing at the national level or higher. Thirteen studies (62%) included more than one sport compared to the eight that only included one specific sport. Of those including only one sport, three (14%) were for rhythmic gymnastics [[23], [24], [25]], two (10%) for volleyball [22,26], and one each for soccer [27], judo [28] and track and field [29]. All studies adopted a quantitative approach, and the majority were of cross-sectional design (n ​= ​18, 86%). Only three studies (14%) adopted a longitudinal design [26,30,31] and there was one randomised controlled trial of an intervention for EDs [30].

**Table 1:** Displays demographic data for the studies that were included in this review. Data displayed were study design, sample size, age, gender, location, the performance level, and the type of sport for the studies included in this review.

Author(s) and year of Publication	Study Design	Sample Size	Age	Gender	Location	Athlete or Sport Level	Sports included
Beals 2002 [22]	Cross-Sectional	23	15.8 ​± ​1.1, range 14–17 years	Girls	USA	National	Volleyball
Boudreault, Gagnon-Girouard 2022 [32]	Cross-Sectional	999	15.68 ​± ​1.16, range 14–17	Boys, Girls	Canada	State & National/International	Multiple
de Oliveira, de Pinho Gonçalves 2017 [23]	Cross-Sectional	Total 27 Included 4 "Child" aged 12, 11 "Juvenile", and 12 "adults"	12-20. subgroups meeting inclusion criteria: "Child" aged 12 ​± ​0, "Juvenile" aged 13.7 ​± ​0.8	Girls	Rio de Janeiro, Brazil	National	Rhythmic Gymnastics
de Sousa Fortes, de Vasconcelos 2017 [26]	Cohort Study	73	15.94 ​± ​1.06, range 15–17	Girls	Pernambuco, Brazil	State	Volleyball
Donti, Donti 2021 [24]	Cross-Sectional	Total 89 41 International level, 48 recreation level	International level: 13.6 ​± ​1.3, Recreational 13.1 ​± ​1. range 13–15.	Girls	Greece	International	Rhythmic Gymnastics
Escobar-Molina, Rodriguez-Ruiz 2015 [28]	Cross-Sectional	Total 144 aged 15–30. 46 "Cadet" aged under 17	15-30. subgroup meeting inclusion criteria: "Cadet" group aged under 17 (15.9 ​± ​0.4)	Boys, Girls	Madrid, Spain	International	Judo
Fortes, Almeida 2014 [29]	Cross-Sectional	88	12–17 (15.0 ​± ​1.7)	Girls	Brazil	Regional or above	Track and Field
Giel, Hermann-Werner 2016 [10]	Cross-Sectional	1138	16.3 ​± ​1.1.	Boys, Girls	Germany	National	Multiple
Martinsen 2010 [33]	Case Control	Total 961 606 Athletes, 355 Controls^**b**^	15-16 (Born in 1992)	Boys, Girls	Norway	Elite Sport Schools (21.8% competed at an international level)	Multiple
Martinsen and Sundgot-Borgen 2013 [34]	Cross-Sectional	Total 966 ​611 Athletes, 355 Controls^**b**^	Athletes- 16.5 ​± ​0.3, Controls 16.9 ​± ​0.3, Born in 1992	Boys, Girls	Norway	Elite Sports School, (17% of athletes were selected for the national team (recruit, junior or senior level))	Multiple
Martinsen, Bahr 2014 [30]	RCT	Total 611 ​330 Intervention group, 247 Control Group^**b**^	No overall mean quoted. Born in 1992	Boys, Girls	Norway	Elite Sport Schools	Multiple
Martinsen, Holme 2014 [31]	Cohort Study	Total 275 Two separate samples, 221^**b**^ Elite Athletes for Phase 1, 53^**b**^ Athletes for Phase III (subset of Phase 1, External group for Phase II included 54 athletes	Phase 1 16.5 ​± ​0.3 Born in 1992 Phase II 16.7 ​± ​0.9 Born in 1992	Girls	Norway	Elite Sport Schools	Multiple
Parlov, Low 2020 [35]	Case Control	Total 70 36 artistic swimmers and 34 female water polo players	Aged 13–16 for both artistic swimmers and water polo groups.	Girls	Croatia	Attended at least one "major sport competition"	Water Polo, Artistic Swimming
Pettersen, Hernaes 2016 [36]	Cross-Sectional	225	Three Age groups. “Aged 17” and “Aged 18” meeting inclusion criteria. Third group “Aged 19+”	Girls	Norway	National	Cross Country Skiers and Biathletes
Pietrowsky and Straub 2008 [21]	Case Control	Total 164 16 Lightweight Rowers aged 15–18, 17 heavyweight rowers aged 15–18 and 16 Handball players aged 15–18.	10-40. subgroup meeting inclusion criteria aged 15–18.	Boys	Germany	National (15–18-year-old subgroup)	Rowing, Handball
Pitil and Wahed 2019 [37]	Cross-Sectional	Total 150 36 aged 13–15 and 71 aged 16-18	10–21 Subgroups meeting inclusion criteria aged 13–15 & 16-18	Girls	Sarawak, Malaysia	National	Multiple
Prather, Hunt 2016 [27]	Cross-Sectional	Total 220 subgroup meeting inclusion criteria: 81 aged 15–17.	10-30. Subgroup meeting inclusion criteria aged 15-17	Girls	USA	National (15–17-year-old subgroup)	Soccer
Rosendahl, Bormann 2009 [38]	Case Control	Total 867 576 Athletes, 291 Non-Athletes	Mean 15.7 for athletes, 15.9 for non-athletes. Range 14–18.	Boys, Girls	Germany	Elite Sports Schools (23 German national athletes, 104 junior national athletes, 348 junior state team athletes, 101 athletes non-belonging to any selection team)	Multiple
Salbach, Klinkowski 2007 [25]	Case Control	Total 164. 50 elite rhythmic gymnasts, 58 AN patients and 56 high-school students	12–18	Girls	Germany	International	Rhythmic Gymnastics
Stornaes, Rosenvinge 2019 [39]	Case Control	Total 832 666 from ordinary secondary schools and 166 students from elite sport schools	13–14	Boys, Girls	Norway	Elite Sports Schools	Not specified
Walter, Heinen 2022 [40]	Cross-Sectional	439	14.9 ​± ​1.4 Range 13-18	Boys, Girls	Germany	Elite Sports School or National	Multiple

**Table 2:** Displays the method of assessment, prevalence rates, risk factors, protective factors and outcomes of disordered eating or eating disorders for the articles included in this review.

Author(s) and year	Method of Assessment	Prevalence rates	Risk Factors Identified	Protective Factors Identified	Outcomes of disordered eating or eating disorders
Beals 2002 [22]	BSQ and the EDI	26% of athletes exceeded the cut off score for BSQ. 35% exceeded the cut of score for the EDI.	Nil	Nil	Reported on menstrual dysfunction but not association with EDI/BSQ.
Boudreault, Gagnon-Girouard 2022 [32]	EWCB measured by asking participant to rate on a four-point Likert scale (0 ​= ​never; 1 ​= ​rarely, 1–2 times; 2 ​= ​sometimes, 3–10 times; 3 ​= ​often, more than 10 times) the frequency with which they have voluntarily used an extreme method (fasting, excessive exercise, use of vomiting, laxatives, diet pills, diuretics) to reach the ideal weight for their sport.	Local/Regional: 17.3%, State 16.0%, National/International 17.7% met criteria for EWCB	Girls, weight class-based sports, Participants with ‘negligent’ coaches or a coach that had perpetrated psychological violence, or negligent parents/stepparents	Nil	Nil
de Oliveira, de Pinho Gonçalves 2017 [23]	EAT-26 & BITE. Severity scale for the BITE was also included.	"Child": EAT-26 0% positive, BITE 0% Positive. "Juvenile": EAT-26 18.2% Positive, BITE 18.2% Medium level, 0% high level. "Adult": EAT-26 16.7% positive, BITE 8.3% High, 18.2% Medium	Nil	Nil	Nil
de Sousa Fortes, de Vasconcelos 2017 [26]	EAT-26 & BSQ	25% of athletes showed risk for onset of disordered eating according to EAT-26 scores.	Increased body fat percentage and coach "Autocratic" subscale of SLS	Coach "Social Support" Subscale of SLS	Nil
Donti, Donti 2021 [24]	EAT-26	41.46% of international level gymnast had an EAT-26 score ≥20 “indicating eating disordered attitudes and behaviours” compared to 14.58% of recreational gymnasts	"Negative reactions to imperfection" as measured by SPQ, increased BMI and training experience.	Nil	Nil
Escobar-Molina, Rodriguez-Ruiz 2015 [28]	EAT-40, FCQ-T, RS	Not directly reported. 5 Cadets were “at risk” for a prevalence calculated by writer of 10.9%. 1 Cadet was considered to have a clinical eating disorder. Prevalence calculated as 2.2%.	Female gender	Increasing age	Girls with eating disorder symptoms had higher anxiety than boys with eating disorder symptoms
Fortes, Almeida 2014 [29]	EAT-26 & BSQ	16% of athletes were considered “at risk of eating disorders” based on cut-off score of EAT-26 of 21.	Nil	Nil	Athletes experiencing high anxiety had lower rates of binge eating and self-induced vomiting. STAI-T was associated with the EAT-26 oral control subscale.
Giel, Hermann-Werner 2016 [10]	FKKS, SIAB-S & SCOFF. “Eating Disorder Pathology” was defined as a) two more SCOFF questions answered affirmatively; b) FKKS subscale body acceptance score <19; c) one or more weight control behaviours reported.	32.5% of the athlete sample fulfilled the criteria for Eating Disorder Pathology.	Weight dependent sports, negative affect, female gender (in those with negative affect). In male athlete's; sport disciplines of endurance, technical or power sports.	Nil	Athletes that were identified as showing Eating Disorder Pathology also scored significantly higher on a screening measure for depression and anxiety compared to athletes without eating disorder pathology.
Martinsen 2010 [33]	EDI-2. Only "drive for thinness" and "body dissatisfaction" subscales were used. Disordered eating defined by one or more of the criteria: drive for thinness score≥15 for girls,≥10 for boys, body dissatisfaction score≥14 for girls,≥10 for boys, BMI<17.9 for girls and <17.5 for boys, trying to lose weight now, tried to lose weight before≥3 times, use of diet pills, laxatives, diuretics or vomiting to reduce weight, self-reported menstrual dysfunction, primary amenorrhea or secondary amenorrhea	Symptoms of Disordered Eating. 44.7% of female athletes, 70.9% of female controls, 13.1% of male athletes, 30.5% of male controls	Female gender, increased BMI	Nil	Nil
Martinsen and Sundgot-Borgen 2013 [34]	Clinical Interview was used to determine DSM-IV eating disorder via EDE. Athletes screened with EDI-2 (only "drive for thinness" and "body dissatisfaction" subscales were included) and a self-report questionnaire on key factors.	Prevalence of DSM-IV Eating Disorder in athletes compared to controls was 7% vs 2.3%. Female athletes vs male athletes was 14% vs 3.2%. 73.5% of athletes with an ED were diagnosed with EDNOS, 23.5% BN and 2.9% AN.	Female gender, dieting, current or previous use of pathogenic weight control methods, younger age of first dieting attempt, reduced training volume in boys, higher BMI in boys, increased drive for thinness in girls, increased body dissatisfaction in females	Nil	Menstrual Dysfunction- 3 athletes with ED had secondary amenorrhoea, 1 had oligomenorrhea out of total of 34.17/34 athletes were above the cut-off for a mental disorder (anxiety/depression, based on SCL-5)
Martinsen, Bahr 2014 [30]	Clinical Interview was used to determine if a participant had DSM-IV eating disorder via EDE. Athletes were screened with EDI-2 (Only subscales "drive for thinness" and "body dissatisfaction" were included) and a self-report questionnaire: training history, physical activity and nutritional patterns, menstrual history, oral contraceptive use, dieting and weight fluctuation history, use of pathogenic weight control methods, injuries, self-report of previous and/or current ED	34 of 611 athletes at pretest had a DSM IV diagnosed ED (5.6%.).	Female gender	Nil	Nil
Martinsen, Holme 2014 [31]	Clinical Interview (EDE), EDI-2, BEDA-Q	Not reported. 28 of 184 female athletes who participated in Phase 1 Clinical Interview had a DSM-IV eating disorder (15.2%).	Dieting (trying to lose weight now or tried to lose weight before≥3 times). Multiple items from the body dissatisfaction, drive for thinness and perfectionism subscales of the EDI were statistically significant predictors of eating disorders.	Nil	Nil
Parlov, Low 2020 [35]	EAT-26 Items with <10% variance were excluded and four behavioural questions added.	2/36 artistic swimmers had EAT-26 Score over 20. Prevalence calculated by writer of 5.6%. Prevalence data for water polo players was not reported.	Leanness sport	Nil	Nil
Pettersen, Hernaes 2016 [36]	EDI-2. Only two subscales were used, DT and BD	Prevalence of disordered eating was 10.4% in the 17-year-old age group and 25.9% in the 18-year-old age group. (The 19+ age group was 20%).	No longer actively competing ^**a**^	Nil	Nil
Pietrowsky and Straub 2008 [21]	FEV-Kind (scale developed by authors), BIA, assessed under both hunger and satiety	Not reported. No cut off score that defined restrained eating or body-dissatisfaction.	Lightweight rowers compared to heavy weight rowers and handball players in those aged 15–18.	Nil	Nil
Pitil and Wahed 2019 [37]	EAT-26	Difficult to interpret as no definition for high-risk or low risk for eating disorder. However, 9 athletes out of 36 aged 13–15 were deemed high risk for eating disorder compared to 4 out of 6 of those aged <12 and 0 for both 16–18 and 19-21 year-old age groups.	Non-weight sports (those that don't have weight categories), Low Body Fat percentage, moderate activity compared to very active.^**a**^	Nil	Nil
Prather, Hunt 2016 [27]	EAT-26. A score of 10–19 indicates an intermediate risk individual and a score of 20 or higher indicates an at-risk individual.	1.9% of high school athletes were considered at-risk for an ED compared to 0% for all other groups. 6.1% of high school athletes were considered intermediate risk compared to the overall average of 7.7%. The College group had the highest percentage of intermediate risk with 17.8%.	Nil	Nil	19% of high school athletes had menstrual dysfunction compared to the overall average of 19.3%. 13.6% of high school athletes had a stress fracture compared to 0.1% for grade/middle school athletes, 7.1% for college and 13.8% for professional athletes.
Rosendahl, Bormann 2009 [38]	EAT-26. Body image and body ideal measured via selecting silhouettes developed by Gutezeit et al. (1986), weight was classified based on BMI compared to German percentile graphs, with only underweight and not underweight considered.	16.3% of the athletes scored 10 or higher on the EAT-26 compared to 26.1% of the non-athletes. Comparing genders, only female athletes had statistically higher EAT scores than non-athletes. Setting the cut-off to 20, 3.5% of athletes were considered at risk for an ED compared to 8.6% of non-athletes.	Female gender. Leanness sport among boys, girls participating in power sports and boys participating in an antigravitation sport all had a higher risk of disordered eating. Non-athletes compared to non-elite athletes had higher rates of disordered eating. The risk for disordered eating was 8 times higher among girls with a thinner body ideal compared to girls with an equal or thicker body ideal.	Nil	Nil
Salbach, Klinkowski 2007 [25]	EDI-2 for children and adolescents (8 subscales were used, excluding ascetism, impulse regulation and social insecurity), The TEK-KJ, these results were reported as BFI. AN patients were assessed with structured interview for diagnosis.	No cut-offs scores were used, and only mean EDI-2 scores reported. AN patients had higher EDI-2 scores on all subscales than both gymnasts and high school students. There was no difference between gymnasts and high school students on all subscales. AN patients scored higher on all four body image BFI scores than both other groups except for the abdomen score compared to gymnasts	Nil	Nil	14% of gymnasts had primary or secondary amenorrhea compared to 5.4% of high school students and 100% of the AN patients
Stornaes, Rosenvinge 2019 [39]	EDE-Q-11. Used to measure WCSC.	Nil	Two personality profiles were identified with higher rates of WCSC. Ordinary school students were more likely to be members of these two profiles than students of elite sports schools^**a**^	One personality profile was found to be protective. Students at elite sports schools were more likely to be members of this personality profile compared to students of ordinary schools^**a**^	Nil
Walter, Heinen 2022 [40]	ChEDE-Q8, ATHLETE Questionnaire & self-report questionnaire: Meals and snacks per day, weight control method that you use regularly	5.5% clinically significant eating pathology. Girls aged 13–14 3.8%, girls 15–18 9.6%. Boys 13–14 0%, boys 15–18 2.6%	In order from most to least explanatory: 1. Sports and Social pressure 2. Personality Factors 3. Sports biography (included age, gender, height, weight, type of sport, training hours, competition level, age at start of career, age at first competition)	Nil	Nil

AN ​= ​Anorexia Nervosa, BD=Body Dissatisfaction, BEDA-Q ​= ​Development of Brief Eating Disorder in Athletes Questionnaire, BFI=Body Feel Indices, BIA=Body Image Assessment, BITE=Bulimic Investigatory Test Edinburgh, BMI=Body Mass Index, BSQ=Body Shape Questionnaire, BN=Bulimia Nervosa, ChEDE-Q8 ​= ​Eating Disorder Examination-Questionnaire for children and youth short-form, DT ​= ​Drive for Thinness, EAT-26 ​= ​Eating Attitudes Test-26, EAT-40 ​= ​Eating Attitudes Test-40, ED ​= ​Eating Disorder, EDE ​= ​Eating Disorder Examination, EDE-Q-11 ​= ​Eating Disorder Examination-Questionnaire, EDI ​= ​Eating Disorder Inventory, EDI-2 ​= ​Eating Disorders Inventory 2, EWCB ​= ​Extreme Weight Control Behaviour, FCQ-T ​= ​Food Craving Questionnaire-Trait, FEV= Fragebogen zum Essverhalten, FKKS=Frankfurt Body Concept Scales, RS=Restraint Scale, SIAB-S=Structured Inventory for Anorexic and Bulimic Disorders, SLS=Sports Leadership Scale, SPQ=Sport Perfectionism Questionnaire, TEK-KJ=Test for Detecting Body Image Distortion in Children and Adolescents, WCSC=Weight and Shape Concern. **a** ​= ​results included athletes or ages not meeting study inclusion criteria for age or elite, **b=**population sampled overlapped to varying degrees.

### 3.2 Measures

Most studies used self-report screening questionnaires to assess for EDs and DE. Nine studies (43%) used variations of the Eating Attitudes Test (EAT), eight (38%) used the EAT-26, whilst one study made use of the EAT-40 [28]. Six studies (29%) utilised variations of the Eating Disorder Inventory-2 (EDI-2) and four studies (19%) used variations of the Eating Disorder Examination (EDE). Three studies (14%) used clinical interview whilst sharing some of the same data [[30], [31], [34]] and three further studies used the Body Shape Questionnaire (BSQ) [22,26,29]. There was a wide variety of alternative screening tools that each appeared in only one study.

### 3.3 Prevalence

The included studies provided prevalence data for a wide range of potential diagnoses. Most commonly, eight of the studies reported on the prevalence of “risk for eating disorder” or a close approximation of this. Five studies (24%) included the diagnosis of DE, and three studies (14%) included the diagnosis of eating disorder meeting DSM criteria [[30], [31], [34]]. There was a variety of other terminology including “clinically significant eating pathology” [40], “Eating Disorder pathology” [10], “Body image disturbances” [22], “Extreme weight control behaviour” [32], “Eating disordered attitudes and behaviours” [24], “restrained eating and body dissatisfaction” [21], and “weight and shape concerns” [39].

Noting the variety of diagnoses, there was a broad range of prevalence data, ranging from 1.9% of athletes “at risk for eating disorder” [27] to 44% of female athletes having “symptoms of DE” [33]. Thirteen studies (62%) reported a prevalence >10% compared to five studies (24%) that reported prevalence <10%. Three studies (14%) did not directly report on prevalence [21,25,39]. Three studies (14%) reported on the prevalence of EDs meeting DSM-IV criteria. Martinsen and Sundgot-Borgen [34] reported a prevalence of 7% in EYAs vs 2.3% for the non-athlete control group, Martinsen, Bahr [30] reported a prevalence of 5.6% in EYAs, and Martinsen, Holme [31] found a prevalence of 15.2% in female EYAs. The most common eating disorder in these studies was Eating Disorder Not Otherwise Specified [[30], [31], [34]].

Regarding the four studies that included non-athlete control groups; two studies reported a higher prevalence amongst controls [33,38], one study reported a higher prevalence amongst the EYAs [34], and one study reported no difference [25]. Only one of these studies used clinical interview [34]. Only one of the studies compared EYAs to recreational or non-elite youth athletes. This study found a much higher prevalence among elite (41%) compared to recreational (16%) gymnasts [24].

### 3.4 Risk and protective factors

Eight studies (38%) identified female gender as a risk factor. Leanness or weight-dependent sports were identified as a risk factor in seven (33%) studies. Five studies (24%) included athletes participating in both leanness and non-leanness sports and found statistically significant increases in the rates of DE or EDs in athletes participating in leanness sports [10,21,40,32,35]. In contrast, both Martinsen, Bratland-Sanda [33] & Martinsen and Sundgot-Borgen [34] found no difference in DE between leanness and non-leanness athletes. Pitil and Wahed [37] even found that athletes competing in non-weight category sports had higher scores on the EAT-26 scale than athletes competing in weight category sports. Increased body mass index (BMI) or body fat percentage was also identified as a risk factor in five studies [24,26,[33], [34],^37^].

Coaching styles were found to be a risk factor in two studies. de Sousa Fortes, de Vasconcelos [26] found that “autocratic” subscale (measured concentration behaviour and imposition of decisions) of Sport Leadership Scale (SLS) explained 8% of the variance of DE behaviour. Boudreault, Gagnon-Girouard [32] found that athletes who had experienced ‘negligent’ (i.e., non-intervening) coaches had 18 times the risk for exhibiting extreme weight control behaviour. Athletes whose coaches perpetrated psychological violence were also 8 times more likely to have utilised extreme weight control behaviour.

One study systematically investigated a broad range of risk factors among youth athletes [40]. A variety of risk factors were identified including sport and social pressure and personality factors such as informed introjection and self-discipline. Media consumption and type of sport played a stronger role among boys whereas personality factors were more significant for girls. Stornaes, Rosenvinge [39] also investigated personality profiles of high school and elite sports school athletes. They found two personality profiles associated with increased rates of “weight and shape concerns”: ‘high mixed perfectionism’ and ‘low self-orientated perfectionism with high perfectionistic concerns. They also found a third profile with higher personal standards and lower external fears and doubts related to performance, who had reduced weight and shape concerns. Students at elite sports schools were more prevalent in the third personality profile and less prevalent in the first two profiles compared to typical students. Donti, Donti [24] found that negative reactions to imperfection was associated with higher EAT-26 scores.

Only three studies (14%) reported on possible protective factors for the development of EDs or DE. These studies showed coach social support [26] increased age [28], and a personality profile described earlier with higher personal standards and lower external fears and doubts [39] found to be protective.

### 3.5 Outcomes of eating disorders or disordered eating

Five studies (24%) investigated the outcomes of EDs or DE, with menstrual dysfunction the most frequently explored [22,25,27,34]. Martinsen and Sundgot-Borgen [34] reported 11.8% of athletes with an ED meeting DSM-IV criteria had menstrual dysfunction. Whereas the remaining studies only provided prevalence of menstrual dysfunction for the entire sample and did not provide data for EYAs meeting criteria for eating disorder or DE [22,25,27].

Three studies (14%) commented on comorbid mental health concerns, though causality was not specifically shown. Martinsen and Sundgot-Borgen [34] showed that half of EYAs with an ED meeting DSM-IV criteria scored above the cut-off for anxiety and depression. Giel, Hermann-Werner [10] showed that athletes fulfilling the study defined criteria for ED pathology scored significantly higher on screening measure for depression than those not meeting criteria for ED pathology. Fortes, Almeida [29] reported that athletes with high anxiety had lower scores on the bulimia and food preoccupation subscale of EAT-26. This is assumed to be an error given this direction for relationship is unexpected along with contrary reporting later in the text of the manuscript (authors contacted via email with no response).

## 4 Discussion

This review set out to summarise what is known about (1) prevalence, (2) risk and protective factors, and (3) outcomes of EDs and DE among EYAs. We identified 21 studies published since 2000, with significant heterogeneity seen across the field, limiting reliable conclusions that can be taken from the literature. The included studies investigated throughout the spectrum of eating behaviour from “symptoms of DE” to an ED meeting DSM-IV criteria. Included populations were heterogenous across genders, locations and types of sport which greatly limited the ability to compare and interpret prevalence data. No studies made use of the updated criteria in DSM-5 [41]. Noting these concerns and as might be expected, prevalence ranged broadly; as high as 44% for symptoms of DE in female high school athletes [33] and as low as 1.9% of high school athletes “at risk for ED” [27]. Perhaps most reliably however, prevalence of athletes with an ED meeting DSM-IV criteria ranged from 5.6% to 7% [30,34]. This is comparatively lower than studies in adult elite athletes (e.g., 13.5% [11] and 28% [42]). Similarly, these rates are considerably lower than the recently reported prevalence ranging from 13.6% to 22.2% of adolescents meeting DSM-5 criteria for an ED among the general population [43]. Previous studies among adult elite athletes have often identified higher rates of EDs than the general population [11,42]. There were mixed findings within the current review, however the only study that used clinical interview for assessment found higher rates among EYAs than non-athletes [34]. Further, the only study that included a non-elite, recreational youth athlete comparison group found that EYAs had nearly triple the prevalence of “eating disordered attitudes and behaviours” [24]. Thus, while further research is necessary to better understand some of these discrepancies, there is preliminary evidence to suggest that EYAs may be at higher risk of DE or EDs than young people not engaged in elite sport.

An important finding from this review is that the included studies utilised at least 15 different self-report screening tools. Most common were the EAT-26 and the EDI-2. This introduces a range of caveats relating to the prevalence data collected. The EAT-26 has demonstrated a reasonable internal consistency reliability in athletes of 0.51–0.93 with respectable consistency in male athletes [44]. However, to our knowledge the EAT-26 has not been validated in youth athletes [45]. There is also a concern that multiple items cater exclusively for a thin ideal internalisation that may impact validity in athletes who instead desire muscularity, impacting recognition across all genders but more commonly boys and men [17,44]. The EDI-2 on the other hand, consists of 11 multidimensional subscales but research has often centred on three of the subscales; “drive for thinness”, “bulimia” and “body dissatisfaction” [46]. It has an internal consistency reliability in adult athletes of 0.69–0.9 and like the EAT-26, has not been validated among EYAs [44]. Frequent modification of the EDI-2 occurred for the papers included in this review. Only 2 subscales, “drive for thinness” and “body dissatisfaction” were used in five of the studies that used the EDI-2 [[30], [31], [33], [34],^36^], while Salbach, Klinkowski [25] made use of the EDI-2 for children and included 8 subscales. In three studies, alterations were made to the questionnaire for male athletes which aimed to screen for body dissatisfaction more accurately [30,34,33]. Despite popularity of the EAT-26 and EDI-2, there were a variety of other tools utilised, such as the Extreme Weight Control Behaviour (EWCB) criteria or Fragebogen zum Essverhalten-Kind [21,32]. There are a variety of screening tools available that are specific to the athlete population, yet only two studies made use of athlete specific questionnaires. Walter, Heinen [40] used the ATHLETE questionnaire and Martinsen, Holme [31] used the Brief Eating Disorder in Athletes Questionnaire in a study that demonstrated its development and validity in female EYAs only. This heterogeneity makes it difficult to compare results between studies and these alterations are not necessarily validated.

Only three studies made use of clinical interview rather than self-report questionnaires. This is despite well-established recognition that clinical interview may be required to accurately determine prevalence of EDs among elite athletes. This is because this population may under-report symptoms, either for fear of jeopardising their athletic careers or because these cognitions and behaviours are rationalised in the context of their sporting environment [11,47]. This same conclusion was also drawn by the included study from Martinsen and Sundgot-Borgen [34] with reduced sensitivity of self-report measures in EYAs compared to controls; 0.85 vs 1.0. Yet, no further studies since 2014 have made use of clinical interview.

With regard to risk and protective factors, female gender was the most cited risk factor with girls shown to have consistently higher prevalence rates compared to boys across all studies that compared genders. Frequently, the prevalence was more than double if not triple for girls, which is consistent with existing literature in adults [11,48]. While acknowledging that screening tools have a bias towards thinness which may inflate gender differences, where clinical interview was utilised, girls still showed a higher prevalence; 14% vs 3.2% [34] & 20.8% vs 2.3% [30]. Although leanness or weight-dependent sports was the next most cited risk factor, this might be partially explained by existing screening tools having bias towards leanness such as “drive for thinness” subscale in the EDI-2 or “I am pre-occupied with a desire to be thinner” as an example item from the EAT-26. Indeed, one of the two studies that found no difference in prevalence between athletes competing in leanness compared to non-leanness sports utilised clinical interview [34,33]. As might have been expected based on existing research [49], increased body mass index or body fat percentage was identified as a risk factor in 5 studies [24,26,[33], [34],^37^]. Typically, this was explained as reflecting increased body dissatisfaction. Finally, coaching style was found to be a key modifiable risk factor [26,32], mirroring findings among adult athletes as an influential contributor to the development of EDs or DE [50].

It is well established that EDs contribute to many serious outcomes across populations, with Anorexia Nervosa having an 18-fold increase in mortality [51]. Yet, few of the included studies in this review investigated outcomes directly. Martinsen and Sundgot-Borgen [34] reported a relatively low prevalence of menstrual dysfunction in EYAs with EDs of 11.8% compared to 16.5% reported in a previous study of elite athletes aged 13–39 [52]. This difference may have been impacted by the way the data was reported, as athletes were identified as either taking oral contraceptives or the presence/absence of menstrual dysfunction [34]. Two studies found an association between EYAs with an ED or DE and increased rates of anxiety or depression [10,34], which is unsurprising as comorbid mental health concerns are common among EDs more broadly [51].

### 4.1 Limitations

Limitations relating to the included studies primarily relate to an over-reliance on quantitative cross-sectional designs, with vastly more studies focusing specifically on girls. This parallels research on EDs in adult athletes which has been primarily focused on women [53], though contrasts with broader trends in sports medicine and psychology [54,55]. Similarly, at least as reported, no study included or identified trans, non-binary or other gendered participants. Emerging evidence suggests higher rates of ED symptoms in transgender youth compared to cis-gender youth [56], and transgender athletes may have higher rates of mental distress [57].

We acknowledge that fundamental decisions in our methodology undoubtedly influence the range of studies included, potentially leading to biases which must be recognised. Specifically, we only searched for studies from 2000 onwards. In our pre-registered protocol, we chose to constrain our review to more current work, with the aim of better capturing recent contextual and environmental factors that relate to the increasing professionalisation of elite youth sport. In doing so however, we may have excluded important work published prior to this date. Further, the included studies were limited to those peer-reviewed and published in English. This may have reduced the reliability of our review through a publication bias towards statistically significant or novel findings. Finally, we did not conduct a risk of bias or study quality assessment. While we acknowledge that these may potentially have been beneficial inclusions, they are neither required for scoping reviews [58], nor are they common in the field of sport psychology [59]. These methodological decisions were informed by a combination of theoretical rationale, previously published guidelines or similar articles, and feasibility constraints within the team.

### 4.2 Future research

Although this review has demonstrated that there is a significant evidence base in this field, a variety of recommendations are provided to advance knowledge through further research. The existing research has focused on prevalence at the expense of better understanding associated factors. Longitudinal studies would allow better understanding of causality regarding risk and protective factors, in addition to subsequent outcomes. Qualitative or mixed methods studies are also needed to better understand the individual experience of these conditions in the elite youth sporting environment. More focus should be placed on the experience and expression of EDs and DE among boys, trans, and other genders as well as other diverse populations who have been largely ignored in this space [53,56,57,60]. Further exploration is needed in populations outside of Europe as most of the included studies in this review were conducted here, and the prevalence of EDs is known to vary by region, frequently lower amongst non-western countries [61].

Critically, future studies should consider using, developing, and consolidating towards population-specific screening tools for EDs and DE. While we recognise the significant practical challenges that exist, the use of clinical interview is the gold standard approach and should be used where possible and whenever there is doubt around the validity of available screening tools in novel populations. This is especially true for athletes with EDs as they are known to under-report symptoms on screening tools [42]. Finally, we note that no studies provided evidence on the management and treatment of these conditions among this population. EDs are difficult to treat with high rates of non-recovery or partial recovery [51] and it seems likely that there are population specific effects in elite youth sport that may impact or benefit treatment and recovery.

## 5 Conclusions

There is preliminary evidence to suggest that EDs or DE may be more prevalent in EYAs compared to non-elite or recreational youth athletes. It seems likely that these disorders are also more prevalent in EYAs compared to the general youth population, however further research is needed to establish if this is reliable. Female gender and leanness sports were the most cited risk factors amongst a multitude of other factors including increased BMI, coaching styles, and perfectionistic personality traits. EDs and DE were shown to be associated with increased rates of anxiety and depression in EYAs, but other associated outcomes were more difficult to interpret and scarcely investigated. The research base is primarily conducted using European, cross-sectional quantitative study designs and typically made use of unvalidated, heterogenous, and self-report screening tools. In addition, the included studies overwhelmingly focused on girls rather than other genders, with studies using screening tools biased towards thin ideal rather than other forms of DE in athletes such as search for muscularity or weight gain. Further high-quality research is needed to better understand the prevalence, risk factors and outcomes of EDs and DE that occur frequently in EYAs.

## Funding

This research did not receive any specific grant from funding agencies in the public, commercial, or not-for-profit sectors. CCW is supported by an Academic Fellowship from the 10.13039/501100007961Melbourne School of Psychological Sciences.

## Author contributions

MM & CW conceptualized the review. All authors provided input the design of the review's protocol. MM conducted the search. MM and HG conducted screening of articles. MM extracted data and led synthesis of findings. MM wrote the first draft of the manuscript. All authors revised the manuscript. CW provided supervision for the project. This project was originally conducted as a Master of Psychiatry thesis at The University of Melbourne by student Dr Maxwell Marrows under supervision from Dr Courtney Walton.

## Declaration of competing interest

All authors declare that they have no conflict of interest.

## Figures and Tables

**Table:** 

**Inclusion Criteria**	
Population	Youth (aged 12–18) elite athletes. Athletes competing in international, national, and regional competitions as well those participating in high performance talent development programs were considered elite
Concept	Eating disorders and disordered eating
Context	Any setting
Types of evidence source	• English Language • Original peer-reviewed journal articles
**Exclusion Criteria**	
	• Full-text not available • Studies prior to January 2000 • Not published in a peer reviewed journal •If the study explores a heterogenous sample (eg. Elite and non-elite youth athletes, or includes child and adult athletes in sample) without providing independent group findings

**Table:** 

**Participant**	Sport∗ OR athlet∗ OR player∗ OR Archer∗ OR badminton OR baseball OR bowl∗ OR softball OR basketball OR boxing OR boxer∗ OR canoe∗ OR cricket∗ OR cycling OR cyclist∗ OR diving OR diver OR equestrian OR fencing OR football∗ OR high jump∗ OR hurdl∗ OR javelin∗ OR judo OR karate OR kayak∗ OR lacrosse OR long jump∗ OR netball∗ OR penthalon OR soccer OR golf OR gymna∗ OR handball∗ OR hockey OR rowing OR rower∗ OR rugby OR running OR sailing OR sailor∗ OR shooting OR shot put∗ OR skate∗ OR skating OR skier∗ OR skiing OR snowboard∗ OR softball OR soccer OR surfing OR swim∗ OR sprint∗ OR squash∗ OR table tennis OR tennis OR taekwondo OR triathl∗ OR volleyball∗ OR water polo OR weight lifting OR wrestl∗	**AND**	elite OR high-performance OR high performance OR professional OR Olympic OR paralympic OR Para OR Wheelchair OR national OR international OR world-class or world class OR highly trained OR competitive OR development∗ OR school OR academy OR Expert OR gift∗ OR talent OR pathway OR institute	**AND**	Adolesc∗ OR child OR young OR youth OR child∗ OR teen OR young people OR young person OR boy∗ OR girl∗ OR student∗ OR junior∗ OR pupil∗
	**AND**				
**Concept**	Eating Disorder∗ OR Disordered Eating OR Anorex∗ OR Anorexia Nervosa OR Bulimi∗ OR Bulimia Nervosa OR Binge-Eating Disorder OR Binge eat∗ OR Relative energy deficiency in sport OR relative energy deficiency OR RED-S OR triad OR female athlete triad OR low energy availability OR LEA OR OSFED OR EDNOS				
	**AND**				
**Context**	None				
